# How Transformational Leadership Shapes Teachers’ Innovative Behavior: A Serial Mediation Model of Creative Growth Mindset and Work Engagement

**DOI:** 10.3390/bs16081321

**Published:** 2026-08-03

**Authors:** Yilin Shen, Yi Li, Kaili Tang, Jia Wu, Qianfeng Li, Shaobei Xiao

**Affiliations:** 1 School of Psychology, Hainan Normal University, Haikou 571158, China; 2 School of Elementary Education, Hainan Normal University, Haikou 571158, China; 3 Adolescent Psychological Development and Education Center of Hainan, Hainan Normal University, Haikou 571158, China

**Keywords:** transformational leadership, teachers’ innovative behavior, creative growth mindset, work engagement, primary and secondary school teachers

## Abstract

Teaching innovation plays a central role in advancing high-quality education and fulfilling the fundamental task of fostering moral development and holistic student growth. However, the internal mechanisms through which transformational leadership is associated with teachers’ innovative behavior remain insufficiently understood. Integrating the implicit theories framework and the job demands–resources (JD-R) model, this study proposes a sequential mediation framework in which transformational leadership is associated with primary and secondary school teachers’ innovative behavior through creative growth mindset and work engagement. Data were collected from 1082 primary and secondary school teachers using validated measures of transformational leadership, creative growth mindset, work engagement, and teachers’ innovative behavior. Structural equation modeling with bootstrapping was employed to test the hypothesized model. The results indicated that transformational leadership was positively associated with teachers’ innovative behavior. Creative growth mindset and work engagement each showed significant indirect associations between transformational leadership and teachers’ innovative behavior. Moreover, the sequential indirect association via creative growth mindset and work engagement was significant, supporting a cognitive–motivational pathway linking transformational leadership to teachers’ innovative behavior. These findings suggest that transformational leadership may relate to teachers’ innovative behavior not only directly but also through a cognitive–motivational pathway involving teachers’ beliefs about the malleability of creativity and their subsequent work engagement. From a practical perspective, these results highlight the potential value of leadership practices that communicate an inspiring vision, support teachers’ growth-oriented beliefs about creativity, and foster sustained motivational engagement.

## 1. Introduction

Teachers’ innovative behavior refers to the intentional generation, promotion, and application of novel ideas in instructional practice and has increasingly been recognized as an important factor contributing to educational improvement and enhanced student learning (Bouwmans et al., 2017). Despite this recognition, many primary and secondary school teachers remain reluctant to move beyond familiar routines, often constrained by concerns about failure, rigid administrative structures, and insufficient resources (Cao et al., 2021; M. Li et al., 2016). Identifying how to effectively foster such behavior is therefore both a pressing practical concern and a significant theoretical question.

Among the factors associated with teachers’ innovative behavior, transformational leadership, as a crucial external resource, has received substantial scholarly attention. Scholars in educational contexts have highlighted the central role of transformational leadership in promoting teacher development, school effectiveness, and instructional improvement (Menon, 2024; Keravnos et al., 2025). Through inspirational motivation, charismatic influence, and the articulation of an innovation-oriented vision, transformational leadership creates a supportive climate that encourages teachers to engage in innovative practices (Bass, 1997; Cao et al., 2021). Empirical studies have documented a positive association between transformational leadership and teachers’ innovative behavior (Lin et al., 2022; Zainal & Mohd Matore, 2021). Researchers have further examined how transformational leadership is associated with subordinates’ innovation motivation and promotes innovative behavior from a motivational perspective, highlighting mediating mechanisms such as self-efficacy and organizational commitment (Cao et al., 2021; Kılınç et al., 2025). Moreover, an empirical study in Cyprus has shown that transformational leadership is positively associated with teachers’ self-efficacy and effectiveness, highlighting the importance of psychological mechanisms in this relationship (Lefteri & Menon, 2025). However, existing studies have primarily focused on motivational or efficacy-based mechanisms, with limited attention to how cognitive belief systems initiate subsequent motivational processes.

Social Cognitive Theory provides an overarching framework for understanding how environmental factors are translated into cognitive, motivational, and behavioral processes (Bandura, 2001). From this perspective, leadership practices shape individuals’ cognitive belief systems, which subsequently guide their motivation and behavior (Schunk & DiBenedetto, 2020). However, Social Cognitive Theory does not fully specify the content of these cognitive beliefs or the mechanisms through which cognition is translated into motivation. To address this limitation, we integrate Dweck’s (2006) implicit theories framework, extended to creativity by Karwowski et al. (2019), with the JD-R model (Bakker & Demerouti, 2017) to explain the cognitive and motivational mechanisms linking transformational leadership to teachers’ innovative behavior. Collectively, evidence from diverse educational systems—including China, Cyprus, and Greece—highlights the importance of transformational leadership in promoting teacher development. Nevertheless, research has paid limited attention to the cognitive and motivational processes through which transformational leadership is associated with teachers’ innovative behavior. Addressing this gap, we integrate Social Cognitive Theory, Dweck’s implicit theories framework as applied to creativity, and the job demands–resources (JD-R) model to propose a sequential cognitive–motivational framework in which transformational leadership is associated with teachers’ innovative behavior through creative growth mindset and work engagement.

### 1.1. Transformational Leadership and Teachers’ Innovative Behavior

Transformational leadership refers to a leadership style that stimulates subordinates’ intrinsic motivation and drives them to achieve performance beyond expectations by conveying high-level values, inspiring creative ideas, and providing support for professional development. Vermeulen et al. (2020) indicated that transformational leaders communicate high expectations, provide developmental opportunities, and encourage teachers to engage in innovation. Otherwise, subordinates who are neglected by their leaders will not only perform poorly as individuals but also harm the overall team performance. Transformational leadership is highly universal and has been proven to effectively enhance organizational and individual performance across multiple fields, including business, education, and healthcare, aligning with the demands for leadership behaviors in complex work environments (Bass & Riggio, 2006). A meta-analysis encompassing 127 global studies found that transformational leadership has had an overall positive effect on employees’ innovative behavior (Koh et al., 2019).

Teachers’ innovative behavior refers to observable teaching practices that teachers carry out in the classroom to solve authentic teaching problems, with the core goal of cultivating students’ creative thinking, ultimately aiming to improve teaching quality and promote student growth (Lambriex-Schmitz et al., 2020; Yan & Liu, 2025). Based on an empirical study of 947 primary and secondary school teachers, Kılınç et al. (2025) found that transformational leadership was positively associated with teachers’ innovative practices (e.g., curriculum adaptation, personalized teaching). Moreover, it can indirectly enhance innovative behavior by fostering teachers’ growth mindset, fully demonstrating the important role of transformational leadership in teachers’ innovative behavior (Kılınç et al., 2025).

According to social exchange theory (Blau, 1964), transformational leaders establish reciprocal relationships with teachers by providing social support, such as emotional care, career development opportunities, and resource access. In turn, teachers may reciprocate by engaging in more proactive innovative behaviors (Vermeulen et al., 2020). First, through firm educational beliefs and professional competence, transformational leaders can earn teachers’ trust and value identification, and serve as role models for teachers’ innovative learning in practice (Al-Husseini et al., 2019). This aspect of transformational leadership, reflected in charismatic influence, may reduce teachers perceived risk of innovation activities, making them more willing to break through the constraints of traditional teaching paradigms and actively explore and apply new teaching methods (Zainal & Mohd Matore, 2021). Second, by constructing a clear and compelling blueprint for educational development, transformational leaders help teachers re-recognize the value of teaching innovation, thereby aligning their personal professional growth with the school’s educational direction and generating stronger intrinsic motivation for innovation (C. Yang & Xu, 2026). Transformational leadership can play an important role in supporting teachers and promoting school improvement under challenging educational conditions (Menon, 2024).

Furthermore, according to the Job Demands–Resources model, the organizational resources provided by transformational leaders (e.g., fostering an innovative climate, offering professional guidance) can effectively alleviate the work pressure teachers face when engaging in teaching innovation, enhance their self-efficacy for innovative practice, and thus facilitate innovative behavior (Bakker & Demerouti, 2017). A large body of empirical research also provides reliable support for this conclusion: a study conducted in a centralized education context found that transformational leadership positively predicted teachers’ innovative behavior, by fostering their growth mindset and long-term orientation toward innovation (Kılınç et al., 2025); a survey by Zainal and Mohd Matore (2021) further showed that school administrators’ transformational leadership behaviors explained 47.0% of the variance in teachers’ innovative behavior. Thus, transformational leadership is an important factor associated with teachers’ innovative behavior. Based on this, the present study proposes the following hypothesis:

**Hypothesis 1.**  *Transformational leadership is positively associated with teachers’ innovative behavior*.

### 1.2. Role of Creative Growth Mindset

In educational settings, the implicit theories framework (Dweck, 2006) provides a foundational framework for understanding teachers’ beliefs about the nature of creativity and its role in shaping motivation and behavior. From this perspective, individuals differ in whether they view abilities as malleable or fixed, which in turn influences their willingness to engage in learning and innovation-related activities. Building on this framework, Karwowski et al. (2019) conceptualized creative mindset as comprising two related but distinct dimensions: creative growth mindset (CGM) and creative fixed mindset (CFM). CGM reflects the belief that creativity can be developed through effort, learning, and practice, whereas CFM reflects the belief that creativity is an innate and relatively unchangeable trait. Empirical evidence suggests that teachers with a stronger growth-oriented belief system are more likely to engage in innovative teaching practices and demonstrate greater openness to adopting new instructional approaches (Liu et al., 2026). Specifically, creative growth mindset may buffer teachers’ concerns about innovation failure, making them more resilient when facing teaching challenges and providing a cognitive foundation for innovative practices (Liu et al., 2026). In contrast, individuals endorsing stronger fixed beliefs tend to perceive creative performance as constrained by innate ability rather than effort, which may reduce their willingness to experiment, take risks, and persist in innovation-related tasks (Karwowski et al., 2019). Such beliefs may therefore inhibit teachers’ engagement in innovative behaviors. Overall, creative mindset represents an important cognitive factor associated with teachers’ innovative behavior, with CGM representing a potentially adaptive pathway and CFM providing a contrasting cognitive perspective.

Based on transformational leadership theory, transformational leaders may promote teachers’ creative growth mindset through inspirational motivation, role modeling, and the communication of a shared vision for innovation (Bass & Riggio, 2006). In educational contexts, transformational leadership, as a key external environmental factor, activates teachers’ growth mindset through multiple mechanisms. For example, through inspirational motivation and the communication of an innovation-oriented vision, leaders may encourage teachers to reconsider traditional teaching practices and explore alternative instructional approaches (Dweck, 2006; Bass, 1997). Kılınç et al. (2025) suggest that leaders’ encouragement of innovation may be associated with teachers’ more adaptive beliefs about creativity. Through inspirational motivation, leaders articulate a school vision of promoting students’ holistic development through innovation, enabling teachers to perceive the connection between their own innovation, professional growth, and school development (Dweck, 2006). Second, leaders provide support tailored to teachers’ innovation needs, such as offering specialized training for teachers lacking experience with digital tools and providing affirmative feedback on innovative teaching plans (Vermeulen et al., 2020). According to implicit theory, teachers who endorse a creative growth mindset are more likely to interpret innovation-related challenges as opportunities for development and translate such beliefs into innovative teaching practices (Scott & Bruce, 1994). In practice, teachers with a creative growth mindset are more likely to integrate novel instructional approaches, experiment with alternative teaching strategies, and continuously refine their practices through reflection and feedback. At the same time, they continuously reflect and adjust—for instance, optimizing innovative plans based on student feedback—thereby refining their teaching practice (Dweck, 2006). Transformational leadership primarily functions as a contextual cognitive–motivational resource that fosters employees’ growth-oriented beliefs (Bass & Riggio, 2006). Accordingly, its influence is expected to be more strongly associated with the enhancement of creative growth mindset, whereas its relationship with creative fixed mindset is likely to be weaker given the latter’s relatively stable and resistant nature. A creative fixed mindset was included as an exploratory contrasting cognitive belief to provide a more comprehensive account of teachers’ creative cognition, rather than as a component of the proposed sequential mediation mechanism. Given the limited understanding of how a creative fixed mindset operates in educational contexts, the present study explored its association with teachers’ innovative behavior without specifying a directional hypothesis. Accordingly, a creative growth mindset was expected to be positively associated with transformational leadership and teachers’ innovative behavior. Based on this rationale, the following hypotheses were proposed:

**Hypothesis 2a.**  *Transformational leadership is positively associated with teachers’ creative growth mindset*.

**Hypothesis 2b.**  *Creative growth mindset is positively associated with teachers’ innovative behavior*.

**Hypothesis 2c.**  *Creative growth mindset mediates the relationship between transformational leadership and teachers’ innovative behavior*.

### 1.3. Role of Work Engagement

Work engagement is an important motivational mechanism linking transformational leadership to teachers’ innovative behavior. According to the Job Demands–Resources (JD–R) Model (Bakker & Demerouti, 2017), work engagement refers to a persistent, positive psychological state that teachers display in their teaching practice, characterized by three core dimensions: vigor, dedication, and absorption (Schaufeli et al., 2002). Work engagement arises from adequate organizational resources and individuals’ perception of work meaningfulness, and this positive psychological state stimulates intrinsic motivation (Bakker et al., 2010). Specifically, teachers with high vigor maintain high energy levels in teaching and demonstrate greater persistence when facing innovation challenges; teachers with high dedication regard teaching innovation as an important manifestation of their professional value and proactively invest extra effort in exploring novel teaching approaches; teachers with high absorption become deeply immersed in teaching improvement, continuously engaging in innovative exploration in areas such as curriculum design and method optimization (Schaufeli & Bakker, 2004).

According to JD-R theory, the support provided by transformational leadership, as a key social resource, can effectively stimulate teachers’ work motivation and thereby enhance their work engagement (Bakker et al., 2008). Recent evidence from Greece likewise demonstrates that transformational leadership positively relates to teachers’ occupational well-being and work-related attitudes, highlighting its importance across educational systems (Panagopoulos et al., 2023). Empirical research has found that transformational leadership significantly increases work engagement among Chinese teachers (Mao et al., 2017). When teachers perceive the value and meaning of their work, their belief that “personal growth aligns with organizational goals” is strengthened, thereby fostering dedication and absorption (Polatcan et al., 2025). Meanwhile, when leaders exemplify high professional competence and innovative spirit, they serve as role models for teachers, stimulating teachers’ work vigor and engagement (Cao et al., 2021). In summary, transformational leadership, particularly through inspirational motivation, charismatic influence, and the articulation of a shared vision, may enhance teachers’ work motivation and work engagement. In turn, teachers with higher engagement are more willing to invest time and effort, proactively confront challenges, and try new methods, which drives them to translate their willingness into teachers’ innovative behavior. Therefore, work engagement plays a significant positive mediating role between transformational leadership and teachers’ innovative behavior. Based on this, the following hypothesis is proposed:

**Hypothesis 3a.**  *Transformational leadership is positively related to teachers’ work engagement*.

**Hypothesis 3b.**  *Work engagement is positively related to teachers’ innovative behavior*.

**Hypothesis 3c.**  *Work engagement mediates the relationship between transformational leadership and teachers’ innovative behavior*.

### 1.4. Creative Growth Mindset and Work Engagement

Implicit theories framework (Dweck, 2006) posits that individuals’ beliefs about the malleability of abilities shape how they interpret challenges and regulate motivation and behavior. In the context of teaching innovation, a creative growth mindset enables teachers to view innovative tasks as opportunities for development rather than evaluations of fixed talent, thereby fostering greater effort investment and behavioral persistence in innovation (Dweck, 2006; Kılınç et al., 2025). From a social cognitive perspective, such cognitive beliefs shape how teachers appraise the value and attainability of innovation-related goals, thereby providing a motivational foundation for subsequent energy investment in work tasks. Building on this cognitive foundation, creative growth mindset functions as a personal cognitive resource that facilitates motivational activation in the form of work engagement, characterized by teachers’ vigor, dedication, and absorption in their work (Schaufeli et al., 2002). Transformational leadership may further reinforce these cognitive evaluations by providing an inspiring vision and innovation-related support, thereby strengthening teachers’ beliefs in the malleability of their capabilities and the value of engaging in innovative teaching practices (Zeng et al., 2019). From the perspective of the Job Demands–Resources (JD-R) theory, such personal cognitive resources are associated with greater motivational energy by strengthening individuals’ capacity to mobilize and sustain effort under demanding work conditions (Bakker & Demerouti, 2017). Consistent with prior empirical evidence, teachers with a growth-oriented belief system are more likely to translate adaptive cognitive appraisals into higher levels of work engagement, particularly in the context of innovation-related demands (Nalipay et al., 2021). Accordingly, a creative growth mindset serves as a cognitive resource that precedes and facilitates motivational activation in the form of work engagement.

**Figure 1:**
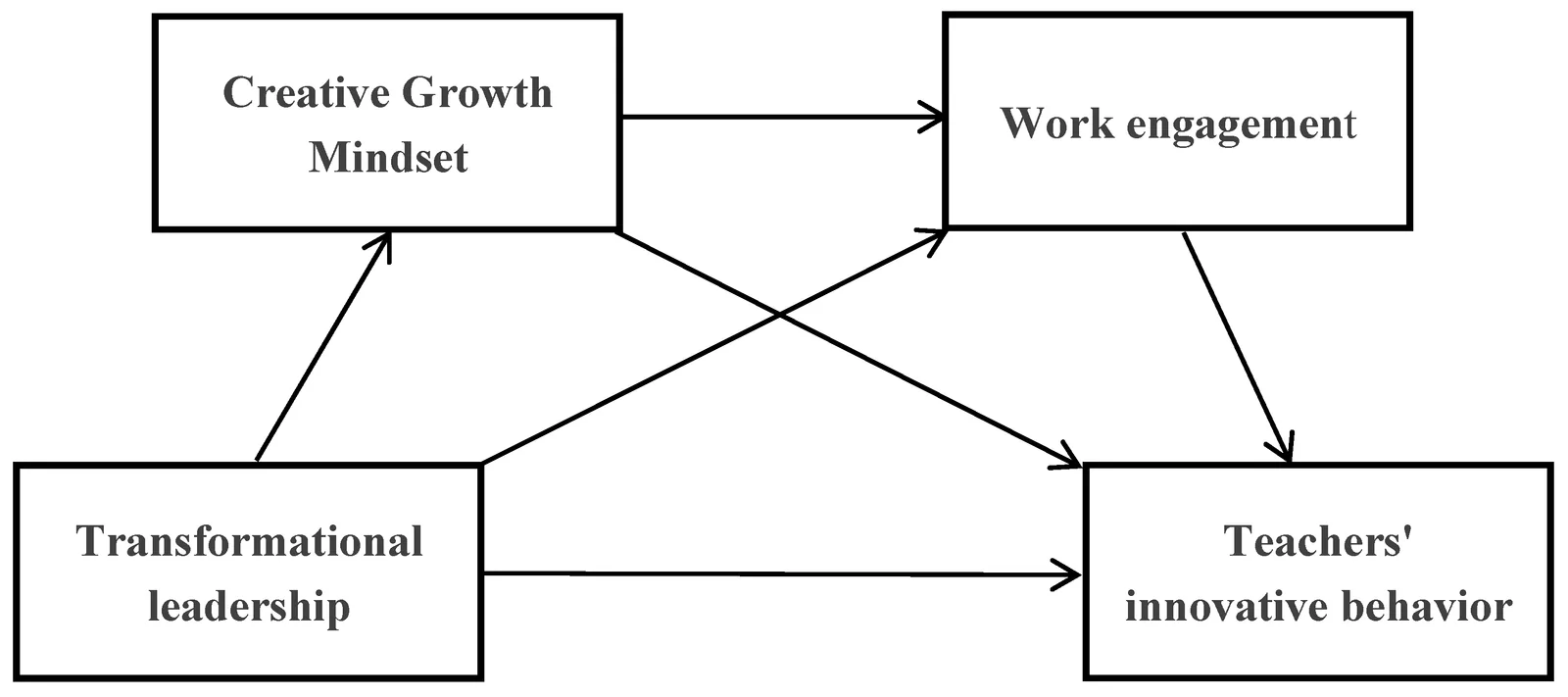
Proposed model.

High work engagement further facilitates the emergence of teachers’ innovative behavior. The JD-R theory provides a mechanism explaining how personal cognitive resources translate into motivational energy under contextual support. According to the JD-R theory, highly engaged teachers translate their intrinsic motivation and psychological resources into concrete practices, contributing to improvements in teaching content, methods, and evaluation (Bakker & Demerouti, 2017). Empirical studies indicate that highly engaged teachers are more inclined to continuously explore novel teaching models and actively participate in teaching reform, thereby contributing to the improvement of educational quality (Yan & Liu, 2025; Su et al., 2022). Integrating implicit theories and the JD-R theory, this sequential pathway from cognition (creative mindset) to state (work engagement) to behavior (teaching innovation) provides a more complete explanation than a single cognitive or affective perspective alone (Bakker & Demerouti, 2017; Dweck, 2006). Taken together, the present study focuses on a sequential cognitive–motivational–behavioral pathway from transformational leadership to creative growth mindset, then to work engagement, and ultimately to teachers’ innovative behavior. Creative fixed mindset is conceptualized as a contrasting cognitive belief to provide a more comprehensive account of teachers’ creative cognition. The research model is shown in Figure 1. Based on this, the following hypothesis is proposed:

**Hypothesis 4.** 
*Transformational leadership is indirectly associated with teachers’ innovative behavior through the sequential mediation of creative growth mindset and work engagement*.

## 2. Materials and Methods

### 2.1. Participants

Participants were 1082 primary and secondary school teachers from Beijing, Henan, Jiangsu, and Chongqing. Participants ranged in age from 22 to 60 years, including 291 male teachers (26.9%) and 791 female teachers (73.1%). Teaching experience ranged from 0 to 41 years, with a mean of 14.73 years.

### 2.2. Procedure

A multi-stage sampling strategy was used to recruit participants from primary and secondary schools. First, convenience sampling was employed to select study regions. Based on the national classification of economic regions in China, four provincial-level regions were selected, including Beijing Municipality and Jiangsu Province representing eastern China, Henan Province representing central China, and Chongqing Municipality representing western China. Subsequently, one county or district was selected from each sampled region for further investigation. Finally, within each selected county or district, schools were recruited through local educational contacts. In larger schools (with more than 60 teachers), approximately 50–70% of teachers were randomly selected from teacher rosters for participation. In smaller schools (with 30–60 teachers), all available teachers were invited to participate. This sampling strategy is commonly used in theory-driven research that aims to examine relationships among psychological constructs rather than estimate population parameters (Creswell & Creswell, 2018). Nevertheless, because the sample was not randomly selected, caution is warranted when generalizing the findings to broader teacher populations. All participants provided informed consent before answering the questionnaire, and then took approximately 15 min to complete the electronic version of the questionnaire. Participants first provided demographic information, including gender, age, teaching experience, educational background, general health status, and sleep quality. They then completed measures assessing transformational leadership, creative mindset, work engagement, and teachers’ innovative behavior. Upon completion, they received 20 RMB as a token of appreciation for their time and participation. The study was approved by the Ethics Committee of Hainan Normal University.

### 2.3. Measures

#### 2.3.1. Transformational Leadership

This study used the revised Chinese version of Transformational Leadership Questionnaire (TLQ) (Bass & Riggio, 2006; Chen et al., 2006). In the present study, transformational leadership was conceptualized as an overall leadership resource reflecting teachers’ global perception of school principals’ transformational behaviors. Accordingly, this study adopted a shortened version of the Transformational Leadership Questionnaire, focusing on two core dimensions—charismatic leadership and inspirational motivation—which capture its central behavioral expressions in school contexts. This approach emphasizes its integrated effect as a contextual resource shaping teachers’ cognitive and motivational processes. The abbreviated scale has been widely used in educational research and has demonstrated acceptable construct validity in Chinese school settings (Chen et al., 2006). Example items include charismatic leadership (e.g., “School leaders are honest, morally virtuous, and lead by example”) and inspirational motivation (e.g., “School leaders articulate a positive vision for the school’s future”). Participants responded to each item on a 6-point Likert scale, ranging from 1 (“strongly disagree”) to 6 (“strongly agree”). The mean score of the scale was used, with higher scores indicating higher levels of perceived transformational leadership of school principals The scale has demonstrated good reliability and validity when applied to Chinese teacher samples. The Cronbach’s α of the TLQ was 0.951.

#### 2.3.2. Creative Mindset

This study used the Chinese version of the Creative Mindset Scale (CMS) (Karwowski et al., 2019; Zhou et al., 2020). The scale contains 10 items, comprising two subscales: creative growth mindset (CGM, e.g., “Everyone is capable of creating something extraordinary when provided with appropriate conditions.”) and creative fixed mindset (CFM, e.g., “You are either inherently creative or not; effort cannot significantly alter this.”), each with 5 items. A 5-point Likert scale was used to measure teachers’ creative mindset, ranging from 1 (“strongly disagree”) to 5 (“strongly agree”). According to Karwowski’s recommendation, CGM and CFM are two closely related but distinct dimensions (Karwowski et al., 2019). Accordingly, CGM and CFM were measured and analyzed as separate variables in the present study. Mean scores were calculated for each dimension, with higher scores indicating stronger endorsement of the respective mindset. This scale has shown good reliability and validity when applied to Chinese teacher samples (Zhou et al., 2020). In the present study, the Cronbach’s α of the CGM was 0.887, and the Cronbach’s α of the CFM was 0.890.

#### 2.3.3. Work Engagement

The Chinese version of Utrecht Work Engagement Scale–Short Form (UWES-9, Schaufeli et al., 2002; F. Li et al., 2013) was used to assess teachers’ work engagement. The scale includes three dimensions, vigor (e.g., “I feel energized when I am working.”), dedication (e.g., “I am enthusiastic about my job.”), and absorption (e.g., “I am immersed in my work.”) each measured by three items, for a total of 9 items. A 6-point Likert scale was used, ranging from 1 (“never”) to 6 (“every day”). The mean score was used, with higher scores indicating higher levels of work engagement. This scale has been validated with good reliability and validity in Chinese populations. The Cronbach’s α of the UWES-9 was 0.944.

#### 2.3.4. Teachers’ Innovative Behavior

The Individual Innovative Behavior Scale (Scott & Bruce, 1994; F. Yang & Zhang, 2012) was used to measure innovative behavior among primary and secondary school teachers. The scale contains six items, and examples of items are “exploring new technologies, processes, methods, and/or new product ideas; generating creative ideas”. A 5-point Likert scale was used to measure teachers’ innovative behavior, with anchors described as 1 = “strongly disagree” to 5 = “strongly agree”. The mean score was used, with higher scores indicating higher levels of teachers’ innovative behavior. This scale has shown good reliability and validity when applied to Chinese teacher samples. The Cronbach’s α of the Individual Innovative Behavior Scale was 0.935.

Finally, all scales used validated Chinese versions that had demonstrated satisfactory psychometric properties in previous studies involving Chinese teacher populations. The factorial validity of all measures was further examined through confirmatory factor analysis.

### 2.4. Data Analysis

As all of the variables in this study were collected by self-reported measures, several procedural controls were adopted during data collection to reduce potential common method bias, including anonymous responding and random ordering of items. After data collection, a post-hoc Harman’s single-factor test was conducted. All scale items were entered into an unrotated exploratory factor analysis. The results extracted multiple factors with eigenvalues greater than 1, with the first factor accounting for 32.08% of the total variance, which did not exceed the critical threshold of 40%. This suggests that common method variance was unlikely to fully account for the observed relationships (Podsakoff et al., 2003). However, because Harman’s test has limited sensitivity, the possibility of common method bias cannot be completely ruled out.

First, descriptive statistics and bivariate correlations among the study variables were examined using IBM SPSS Statistics Version 23.0. Second, confirmatory factor analysis (CFA) was conducted to examine the construct validity of the measurement model. Third, structural equation modeling (SEM) was employed to test the hypothesized sequential mediation model. This approach allows for the simultaneous estimation of measurement and structural components while accounting for measurement error in latent variables. All CFA and SEM analyses were conducted using Mplus Version 8.0.

For the measurement model, CFA was estimated using the maximum likelihood robust (MLR) estimator. Examination of skewness and kurtosis indicated slight departures from normality (skewness ranged from −0.588 to −0.124; kurtosis ranged from −0.168 to 2.207). Therefore, the robust MLR estimator was adopted for CFA to obtain reliable parameter estimates under non-normality. The structural model was estimated using maximum likelihood (ML) estimation. Indirect associations were examined using bias-corrected bootstrap confidence intervals based on 5000 resamples. If the 95% CI did not include zero, the indirect effect was considered statistically significant (Cheung & Lau, 2008). No missing data were observed for any analyzed variables; therefore, full information maximum likelihood (FIML) was not required.

Model fit was evaluated using multiple indices, including the chi-square statistic (χ^2^), comparative fit index (CFI), Tucker–Lewis index (TLI), root mean square error of approximation (RMSEA), and standardized root mean square residual (SRMR). Model fit was considered acceptable when CFI and TLI values exceeded 0.90, RMSEA was below 0.08, and SRMR was below 0.08 (Kline, 2016). Given that χ^2^ is sensitive to sample size, particularly in large samples, model adequacy was evaluated based on the overall pattern of fit indices rather than χ^2^ alone. Indirect effects were considered statistically significant when the 95% bias-corrected bootstrap confidence interval did not include zero.

## 3. Results

### 3.1. Descriptive Statistics

Table 1 presents the descriptive statistics for all variables in the study. This study included a total of 1082 primary and secondary school teachers as participants. The mean age of participants was 37.18 years (SD = 8.56), of whom 73.1% were female and 26.9% were male. In terms of administrative positions, 87.4% of the teachers held no administrative roles, whereas 2.8% and 9.8% held school-level and middle-level administrative positions, respectively. Regarding educational background, the majority held a bachelor’s degree (76.9%), 14.3% held a postgraduate degree or above, and 8.5% held a junior college degree or lower.

In terms of health and sleep status, 39.6% of the teachers rated their general health status as “moderate,” 31.0% as “relatively good,” and 13.5% as “very good,” while 15.9% rated their health as poor or very poor. For sleep quality, 39.9% rated it as “moderate,” 23.3% as “relatively good,” and 9.1% as “very good,” while 27.7% rated their sleep quality as poor or very poor.

Descriptive statistics for the core study variables were as follows: the mean score for transformational leadership was 4.50 (SD = 0.86), for creative growth mindset was 3.69 (SD = 0.60), for creative fixed mindset was 3.13 (SD = 0.78), for work engagement was 4.29 (SD = 1.12), and for teachers’ innovative behavior was 3.85 (SD = 0.56).

### 3.2. Correlations

Table 2 presents Pearson correlation coefficients for all variables in the study. The results indicated that transformational leadership was significantly positively correlated with creative growth mindset (*r* = 0.327, *p* < 0.001), work engagement (*r* = 0.407, *p* < 0.001), and teachers’ innovative behavior (*r* = 0.406, *p* < 0.001). Creative growth mindset was significantly positively correlated with work engagement (*r* = 0.401, *p* < 0.001) and teachers’ innovative behavior (*r* = 0.478, *p* < 0.001). Work engagement was significantly positively correlated with teachers’ innovative behavior (*r* = 0.582, *p* < 0.001). As an exploratory analysis, the association between a creative fixed mindset and teachers’ innovative behavior was examined. A creative fixed mindset was not significantly associated with transformational leadership (*r* = −0.007, ns), whereas it was positively associated with teachers’ innovative behavior (*r* = 0.100, *p* < 0.01). Consistent with the theoretical positioning of the present study, which focuses on the cognitive–motivational pathway involving creative growth mindset, CFM was examined as a contrasting cognitive belief and was not included in the sequential mediation SEM model.

### 3.3. Sequential Mediation Analysis

A confirmatory factor analysis (CFA) was conducted to examine the factorial validity of the measurement model. The hypothesized four-factor model (transformational leadership, creative growth mindset, work engagement, and teachers’ innovative behavior) demonstrated an acceptable fit to the data: χ^2^/df =7.211, RMSEA = 0.076, CFI = 0.922, TLI = 0.913, and SRMR = 0.039. The confirmatory factor analysis indicated that the four-factor model demonstrated an acceptable fit to the data, supporting the distinctiveness of the constructs. Therefore, we proceeded to test the structural model using SEM.

**Figure 2:**
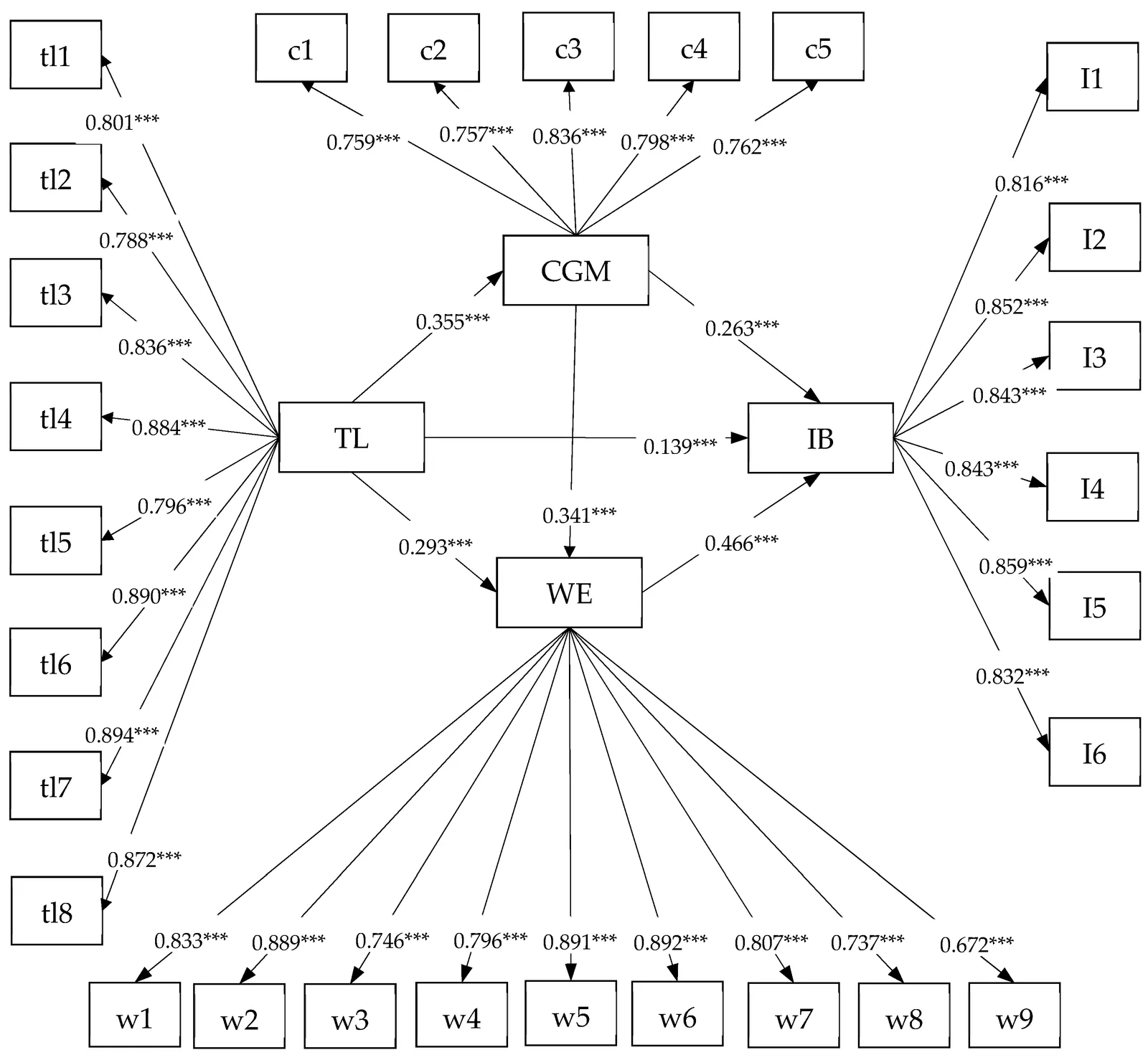
Structural equation modeling analysis. Note: *** *p* < 0.001. TL = transformational leadership; CGM = creative growth mindset; WE = work engagement; IB = innovative behavior.

Structural equation modeling was conducted to examine the hypothesized sequential mediation model. The structural model demonstrated an acceptable fit to the data, χ^2^/df = 6.062, RMSEA = 0.068, CFI = 0.916, TLI = 0.908, and SRMR = 0.059. After including demographic variables as covariates, the SEM results indicated that transformational leadership was positively associated with teachers’ innovative behavior (*β* = 0.139, *p* < 0.001). Transformational leadership was also positively associated with creative growth mindset (*β* = 0.355, *p* < 0.001) and work engagement (*β* = 0.293, *p* < 0.001). Creative growth mindset was positively associated with work engagement (*β* = 0.341, *p* < 0.001) and teachers’ innovative behavior (*β* = 0.263, *p* < 0.001). In addition, work engagement was positively associated with teachers’ innovative behavior (*β* = 0.466, *p* < 0.001). These findings indicate that the proposed sequential cognitive–motivational model was supported. The results are presented in Figure 2.

As shown in Table 3, indirect effects were tested using bias-corrected bootstrap confidence intervals based on 5000 resamples. The results showed that the direct association between transformational leadership and innovative behavior was significant (estimate = 0.099, SE = 0.026, 95% CI [0.053, 0.154]), the indirect association between transformational leadership and innovative behavior via creative growth mindset was significant (estimate = 0.067, SE = 0.013, 95% CI [0.045, 0.094]). Similarly, the indirect effect via work engagement was significant (estimate = 0.097, SE = 0.015, 95% CI [0.071, 0.130]). Importantly, the sequential mediation pathway from transformational leadership to innovative behavior via creative growth mindset and work engagement was also significant (estimate = 0.040, SE = 0.007, 95% CI [0.029, 0.056]), as the confidence interval did not include zero. These findings support the hypothesized sequential cognitive–motivational mechanism, suggesting that transformational leadership is associated with teachers’ innovative behavior through a sequential pathway involving creative growth mindset and work engagement.

## 4. Discussion

Based on Social Cognitive Theory, the implicit theories framework, and the job demands–resources (JD-R) model, this study explores the cognitive and motivational mechanisms through which transformational leadership is associated with teachers’ innovative behavior. Three key findings were identified: transformational leadership is positively associated with teachers’ innovative behavior; creative growth mindset mediated this association; and creative growth mindset and work engagement sequentially mediated the relationship between transformational leadership and teachers’ innovative behavior. Collectively, these results reveal the complete pathway by which transformational leadership activates individual psychological resources and drives the transition from cognition to behavior, providing integrated empirical evidence for understanding how school management stimulates teachers’ innovative behavior.

First, the results showed that transformational leadership was directly and positively associated with teachers’ innovative behavior. School administrators’ transformational leadership practices may provide support and motivation associated with teachers’ innovative behavior, which is consistent with Bass’s (1997) core proposition that transformational leadership inspires subordinates to exceed expectations, as well as the findings of Zainal and Mohd Matore (2021) focusing on teachers. Consistent evidence from European primary and secondary schools further confirms that transformational leadership is associated with teachers’ positive work attitudes and organizational identification (Zacharo et al., 2018). On the one hand, through their charisma and developmental vision, transformational leaders enhance teachers’ identification with the value of innovation (Kılınç et al., 2025). When teachers recognize the educational value of teaching innovation and its significance for professional development, they internalize innovative practices as their own professional goals and responsibilities, actively break away from traditional, stability-seeking teaching models, and translate their belief in the value of innovation into concrete actions: integrating interdisciplinary elements into curriculum design, trying inquiry-based and project-based teaching models in classroom implementation, and continuously optimizing teaching plans through after-class reflection, ultimately promoting the realization of teachers’ innovative behavior. On the other hand, through the resource support and cognitive guidance they provide, transformational leaders reduce teachers’ perceived risk of innovation and stimulate their innovation willingness (Vermeulen et al., 2020). Educational administrators may promote teacher innovation by strengthening transformational leadership behaviors reflected in inspirational motivation, charismatic influence, and the articulation of an innovation-oriented vision. For example, “teaching innovation workshops” could be regularly organized to provide teachers with a platform for communication and exploration (Bass, 1997; Vermeulen et al., 2020), thereby facilitating the implementation of transformational leadership in school settings.

Second, the present study found that creative growth mindset mediated the relationship between transformational leadership and teachers’ innovative behavior; that is, transformational leadership may be indirectly associated with teachers’ innovative behavior through its relationship with teachers’ creative growth mindset. This finding reveals a potential mechanism linking transformational leadership to teaching innovation (Dweck, 2006; Kılınç et al., 2025). Through inspirational motivation and charismatic influence, transformational leaders may provide teachers with encouragement and support for experimentation, enabling teachers to believe that teaching innovation abilities can be developed through continuous learning and reflective practice, thereby cultivating a growth mindset (Kılınç et al., 2025). Moreover, teachers with a growth mindset perceive challenges in teaching innovation as opportunities for ability development rather than as denials of their own competence; therefore, they are more willing to invest time and effort in exploring innovative practices such as interdisciplinary teaching and digital instruction (Liu et al., 2026). Thus, transformational leadership may be associated with teachers’ innovative behavior through the development of growth-oriented creative beliefs. School administrators can stimulate teachers’ interest in creativity through inspirational motivation and create an environment that encourages trial and error, further advancing teachers’ innovative behavior. In addition, the non-significant association between transformational leadership and creative fixed mindset suggests that fixed beliefs about creativity may function as relatively stable cognitive schemas that are less susceptible to contextual influence. Nevertheless, although previous studies have often conceptualized fixed beliefs as potentially constraining creativity-related behaviors (Karwowski et al., 2019), the present exploratory finding revealed a small but significant positive association between creative fixed mindset and teachers’ innovative behavior. This unexpected pattern suggests that the role of creative fixed mindset may be more complex and context-dependent among teachers. One possible explanation is that teachers who perceive creativity as a relatively stable personal characteristic may still engage in innovative practices when they possess sufficient professional motivation, organizational support, or external expectations for innovation. In educational settings, such beliefs may not necessarily prevent innovation but may influence how teachers interpret and approach innovative tasks. Future research should further examine the boundary conditions under which creative fixed mindset may facilitate or hinder innovative behavior.

Most importantly, creative growth mindset and work engagement demonstrated a significant sequential mediation effect between transformational leadership and teachers’ innovative behavior. This finding supports the Job Demands–Resources model, which posits that organizational resources activate individuals’ positive work states, thereby facilitating goal-directed behaviors (Bakker & Demerouti, 2017; Schaufeli et al., 2002). The results reflect a cognitive-to-behavioral transformation process (Nalipay et al., 2021). Overall, the independent mediating effects of creative growth mindset and work engagement, together with their chain mediating effect, accounted for a substantial proportion of the total association between transformational leadership and teachers’ innovative behavior. On the one hand, teacher innovation is a process from cognition to behavior; a single mediator can only explain part of the mechanism, whereas the combination of chain mediation and independent mediation more fully captures the role of transformational leadership, offering greater explanatory power. On the other hand, work engagement serves as a key link between psychology and behavior, directly translating cognitive resources into innovative practice, and represented an important motivational pathway (Cao et al., 2021; Ma, 2023). This mediating function of work engagement between organizational resources and innovative behavior is also verified in higher education contexts, which supports the cross-stage generalizability of this pathway (Hassan et al., 2024). Thus, school administrators should optimize transformational leadership behaviors, cultivate teachers’ growth mindset, and enhance teachers’ work engagement to further stimulate teachers’ innovative behavior (Lin et al., 2022).

The present study has the following three limitations. First, a cross-sectional survey design was used for data collection, which can only capture variable states at a single time point. Consequently, it is difficult to clearly delineate the dynamic relationships among transformational leadership, creative growth mindset, work engagement, and teachers’ innovative behavior, and causal inferences cannot be drawn from the present findings. Future research could adopt longitudinal designs to collect data over extended periods, observe the interplay among variables, and further test causal relationships. Second, all data were collected through teacher self-reports. Although Harman’s single-factor test did not indicate a dominant common method factor, this procedure alone cannot rule out the possibility of common method variance. Because all variables were obtained from the same respondents using self-report questionnaires, shared method variance may have inflated the observed associations. Therefore, future studies are encouraged to adopt multi-source, multi-wave, or experimental designs to reduce common method bias and strengthen causal inference. Third, this study only focused on positive psychological resources and behaviors such as transformational leadership, creative growth mindset, and work engagement, and did not incorporate negative factors that may affect teachers’ innovative behavior, such as teacher burnout, teaching pressure, and innovation anxiety. Meanwhile, the study did not fully explore the differences in resource allocation, innovation climate, and support systems between urban and rural schools. The present study did not examine whether these factors moderated the relationships among variables, nor did it elaborate on the differentiated impacts of transformational leadership mechanisms across different backgrounds. Therefore, Future research could incorporate teachers’ negative emotional experiences as potential moderators to examine whether the proposed sequential mediation process varies across different emotional contexts. Finally, although the present study employed a validated abbreviated TLQ focusing on charismatic leadership and inspirational motivation, transformational leadership is a multidimensional construct. Therefore, future research should adopt the full transformational leadership scale to examine whether other dimensions, such as intellectual stimulation and individualized consideration, contribute differently to teachers’ innovative behavior.

## 5. Conclusions

The purpose of this study was to investigate the mechanisms through which school administrators’ transformational leadership is associated with teachers’ innovative behavior. The results showed that transformational leadership was not only directly associated with teachers’ innovative behavior but was also indirectly associated with it through two important pathways: first, through its association with creative growth mindset, reflecting teachers’ beliefs that their innovative abilities can be continuously improved; second, through its association with work engagement, reflecting teachers’ energy and willingness to invest effort in their teaching. Consistent with recent European research (Keravnos et al., 2025), transformational leadership has been shown to enhance teacher trust and school-level outcomes. The present study extends this line of work by proposing a sequential cognitive–motivational mechanism. Moreover, transformational leadership may first be associated with more positive cognitive beliefs about innovation among teachers, which may subsequently relate to greater work engagement and stronger willingness to engage in innovative teaching practices, optimize classroom design, and improve instructional approaches. Extending prior findings that have primarily focused on organizational outcomes, the present study highlights individual-level cognitive and motivational mechanisms.

In the future, school administrators may adopt transformational leadership characterized by inspirational motivation, charismatic influence, and encouragement of experimentation and innovation, while reducing rigid, controlling management styles to promote frontline teachers’ creative growth mindset and teaching innovation. Schools may also create supportive environments that strengthen teachers’ growth-oriented creative beliefs and enhance work engagement through resource provision and recognition of teachers’ professional value. When leadership style, teachers’ cognitive beliefs, and work states work in concert, teachers’ innovative behavior can be more effectively stimulated, thereby providing stable momentum for improving educational quality.

## Figures and Tables

**Table 1:** Description of demographic variables.

		Number	Percentage	M	SD
Overall		1082	100%		
Age (Mean, SD)				37.18	8.56
Gender	Female	791	73.1%		
	Male	291	26.9%		
Administrative position	School-level cadres	30	2.8%		
	Middle-level cadres	106	9.8%		
	None	946	87.4%		
	Technical Secondary School or High School	11	1%		
	Junior College	81	7.5%		
Educational background	Bachelor’s Degree	832	76.9%		
	Postgraduate Degree and Above	155	14.3%		
	Others	3	0.3%		
	Very Poor	37	3.4%		
	Poor	135	12.5%		
General health status	Moderate	429	39.6%		
	Relatively Good	335	31%		
	Very Good	146	13.5%		
	Very Poor	101	9.3%		
Sleep quality	Poor	199	18.4%		
	Moderate	432	39.9%		
	Relatively Good	252	23.3%		
	Very Good	98	9.1%		
Transformational leadership				4.50	0.86
Creative growth mindset				3.69	0.60
Creative fixed mindset				3.13	0.78
Work engagement				4.29	1.12
Teachers’ innovative behavior				3.85	0.56

**Table 2:** Correlation Analysis.

	(1)	(2)	(3)	(4)	(5)	(6)	(7)	(8)	(9)
(1) Gender	-								
(2) Educational Background	0.168 ***	-							
(3) Administrative position	0.292 ***	0.070 *	-						
(4) General health status	−0.071 *	0.008	−0.074 *	-					
(5) Sleep quality	−0.020	0.074 *	−0.054	0.678 ***	-				
(6) Transformational leadership	−0.019	0.034	−0.095 **	0.144 ***	0.157 ***	-			
(7) Creative growth mindset	0.014	−0.012	−0.059	0.123 ***	0.124 **	0.327 ***	-		
(8) Creative fixed mindset	−0.012	0.016	0.022	0.025	0.004	−0.007	0.239 ***	-	
(9) Work engagement	−0.010	−0.062 *	−0.058	0.208 ***	0.198 ***	0.407 ***	0.401 ***	0.059	-
(10) Teachers’ innovative behavior	0.016	0.004	−0.103 **	0.156 ***	0.119 ***	0.406 ***	0.478 ***	0.100 **	0.582 ***

Note: * *p* < 0.05, ** *p* < 0.01, *** *p* < 0.001.

**Table 3:** Mediation Effect Analysis.

Model Paths	Parameter Estimate	SE	Bias-Corrected CI (95%)
TL → IB	0.099	0.026	[0.053, 0.154]
TL → CGM → IB	0.067	0.013	[0.045, 0.094]
TL → WE → IB	0.097	0.015	[0.071, 0.130]
TL → CGM → WE → IB	0.040	0.007	[0.029, 0.056]
Total effect	0.304	0.034	[0.243, 0.374]

Note: TL = transformational leadership; CGM = creative growth mindset; WE = work engagement; IB = innovative behavior.

## Data Availability

The data presented in this study are available on request from the corresponding author due to ethical and institutional restrictions.

## Abbreviations

The following abbreviations are used in this manuscript:

JD-R	Job Demands–Resources
CFM	Creative Fixed Mindset
CGM	Creative Growth Mindset
TLQ	Transformational Leadership Questionnaire
CMS	Creative Mindset Scale
UWES-9	Utrecht Work Engagement Scale–Short Form
